# Survival and disease burden analyses of occupational pneumoconiosis during 1958–2021 in Huangshi city, China: a retrospective cohort study

**DOI:** 10.1186/s12889-024-18847-6

**Published:** 2024-05-29

**Authors:** Hai-Lian Chen, Chun-Hu Li, Pei-Yao Zhai, Xun Zhuang, Yu-Long Lian, Xue Qiao, Jian Feng, Zu-Shu Qian, Gang Qin

**Affiliations:** 1 Joint Division of Clinical Epidemiology, Affiliated Hospital of Nantong University, School of Public Health of Nantong University, Nantong, Jiangsu China; 2 Huangshi Center for Disease Control and Prevention, Huangshi, Hubei China; 3 Department of Infectious Diseases, Affiliated Hospital of Nantong University, Medical School of Nantong University, Nantong, Jiangsu China; 4 Department of Epidemiology and Biostatistics, School of Public Health of Nantong University, Nantong, Jiangsu China; 5 National Key Clinical Construction Specialty-Department of Respiratory and Critical Care Medicine, Affiliated Hospital of Nantong University, Nantong, Jiangsu China

**Keywords:** Pneumoconiosis, Survival analysis, Disability-adjusted life years, Competing risks model

## Abstract

**Background:**

Pneumoconiosis, a chronic disease stemming from prolonged inhalation of dust particles, stands as a significant global burden of occupational diseases. This study aims to investigate the survival outcomes of pneumoconiosis patients in Huangshi city, China, while also evaluating the disease burden on afflicted patients.

**Methods:**

Data for this study were sourced from the Huangshi Center for Disease Control and Prevention. Survival analyses of pneumoconiosis patients were conducted employing life tables and the Kaplan-Meier method. The Cox proportional hazards models were deployed to identify factors influencing pneumoconiosis patients’ survival duration. Competing risks models were employed to confirm the validity of the model outcomes. Additionally, in the disease burden assessment, disability-adjusted life years (DALYs) were computed for various demographic groups and time frames.

**Results:**

A total of 5,641 pneumoconiosis cases, diagnosed in Huangshi City, Hubei Province between 1958 and 2021, were incorporated into the cohort analysis. The probability of mortality and the risk ratio increased with advancing age. Notably, the median survival time of stage III pneumoconiosis patients was significantly shorter compared with those in stages I and II. The Cox proportional hazards model and competing risks analyses underscored several significant factors influencing survival time, including dust exposure duration (HR = 1.197, 95% CI: 1.104–1.298), age at first diagnosis (HR = 3.149, 95% CI: 2.961–3.349), presence of silicosis (HR = 1.378, 95% CI: 1.254–1.515), and stage II-III pneumoconiosis (HR = 1.456, 95% CI: 1.148–1.848). Cumulatively, DALYs amounted to 7,974.35 person-years, with an average of 1.41 person-years. The period between 2000 and 2019 witnessed the highest disease burden.

**Conclusion:**

Our findings highlight the urgent need for improved prevention, earlier detection, and more effective management strategies for the occupational pneumoconiosis population. This study not only underscores the persistent issue of pneumoconiosis in industrial environments but also serves as a crucial call to action for policymakers and healthcare providers.

**Supplementary Information:**

The online version contains supplementary material available at 10.1186/s12889-024-18847-6.

## Introduction

Pneumoconiosis is a chronic occupational disease caused by long-term inhalation of dust particles, and it is a major public health problem globally [1]. In developing countries where coal is the primary source of energy, millions of workers are exposed to coal dust during their occupational activities. Coal worker pneumoconiosis (CWP) constitutes a significant portion of occupational diseases [2]. As of 2021, there have been reported 1.025 million cases of occupational diseases nationwide, with 915,000 cases attributed to pneumoconiosis, and nearly half of them being CWP. The number of new CWP cases remains alarmingly high each year [3]. Common clinical symptoms of pneumoconiosis include coughing, difficulty breathing, phlegm production and chest pain. Currently, there is no effective treatment for the lung damage caused by pneumoconiosis, and treatment mainly involves symptomatic and rehabilitative care [1]. As a lifelong occupational disease, pneumoconiosis leads to reduced patient lifespan and workability, causing significant health and economic losses [4].

Over the recent decades, China has emerged as one of the nations most impacted by industrial dust exposure on a global scale. Amidst its swift economic expansion and surging demand for mineral resources, an array of smaller mines and smelting operations across the country continues to rely on archaic production methods, suffer from a scarcity of proper protective gear, and grapple with subpar health oversight [5]. Consequently, China stands as one of the most heavily afflicted countries by occupational dust hazards internationally, evidenced by its substantial number of pneumoconiosis sufferers and the considerable portion of the populace subjected to dust exposure [6, 7]. According to national prevalence assessments, there were approximately 525,000 cases of pneumoconiosis in China from 1949 to 1996, increasing to 600,000 cases by 2000 [8]. According to data released by the National Health Commission, in 2019 there were 15,898 new cases of pneumoconiosis in China, which represents a significant decrease compared to previous years [9]. Disability adjusted life years (DALYs), as an indicator of disease burden, can be used to compare the health losses of different regions and populations, to provide a vital basis for the rational allocation of health resources and the evaluation of health policies [10]. In 2017, the global total DALYs attributable to occupational pneumoconiosis was 507,425 person-years, of which China accounted for 48.80% (247,619 person-years) [11]. In a study conducted in Jiangsu Province, the cumulative DALYs caused by pneumoconiosis was 154,500.83 person-years in 1956–2021 [12]. According to Cynthia Lu et al., the estimated prevalence of coal workers’ pneumoconiosis in the United States at the beginning of the 21^st^ century exceeded that of the 1990s [13]. It can be seen that pneumoconiosis has brought significant harm both domestically and internationally that cannot be ignored.

In an effort to prevent pneumoconiosis, the International Labor Organization (ILO) and the World Health Organization (WHO) launched a global program to eliminate silicosis by 2030 [14]. In 2016, China released the National Occupational Disease Control Plan (2016–2020), aiming to promote public health and further protect laborers’ rights. Although the government has made significant efforts, pneumoconiosis remains one of the major social burdens.

Huangshi city, situated in Hubei Province of China, boasts abundant mineral resources, particularly in the fields of metallurgy, coal, and cement materials. The surge in mineral-related industries resulted in a significant rise in pneumoconiosis cases in Huangshi city, making it the primary area for such cases within Hubei Province [15]. Therefore, the prevention and treatment of pneumoconiosis in this area are of paramount importance. We analyzed data on patients diagnosed with occupational pneumoconiosis from 1958 to 2019 in Huangshi city. The aim of this study was to investigate the epidemiologic characteristics and survival status of patients with pneumoconiosis in Huangshi city, as well as to identify the risk factors affecting patient survival. The study further explored the disease burden of pneumoconiosis and its temporal trends, providing an important basis for designing comprehensive prevention and control measures for occupational pneumoconiosis.

## Methods

### Study setting and population

Huangshi city is composed of six administrative districts. The patient data incorporated in this research were sourced from a range of medical units across these districts. Specifically, the data pertaining to pneumoconiosis patients utilized in this study were systematically gathered from a variety of mining and industrial operations within Huangshi city, encompassing coal mines, metal mining sites, cement production facilities, among others, as detailed in Figure S1.

Inclusion criteria: (1) The patients must have been diagnosed by a qualified institution in Huangshi city, with the diagnosis confirmed by at least three radiologists in accordance with the “Diagnostic Criteria for Pneumoconiosis”; (2) Follow-up duration of at least 2 years, or until death, or up to December 31, 2021, with initial exclusions based on these criteria; (3) Comprehensive records, including data on age at birth and history of environmental exposure.

Cause of death encompassed fatalities resulting from pneumoconiosis or its associated complications, including pulmonary heart disease, tuberculosis, and others. Censored cases were delineated as individuals whose demise stemmed from alternate causes, such as accidents, liver failure, renal and autoimmune diseases, among others, or those who remained alive at the conclusion of the observation period.

### Diagnostic criteria of pneumoconiosis

The Occupational Disease Control Section of the Centers for Disease Control and Prevention (CDC) conducts annual follow-up on cases of pneumoconiosis for up-to-date data. Pneumoconiosis diagnosis adheres to diagnostic criteria agreed upon by no less than three radiologists. The diagnostic criteria for pneumoconiosis spanning 1956–2021 are outlined below: “Medical Preventive Measures for Silica Dust Workers”, “GB5906-1986 Dust Lung X-ray Diagnosis Standard and Treatment Principles”, “GB5906-1997 Dust Lung X-ray Diagnosis”, “GBZ 70-2002 Pneumoconiosis Diagnostic Standard 13”, “GBZ 70-2009 Diagnostic Standard 14 and Pneumoconiosis Diagnostic Standard 12 in 2015”. All procedures in this study were approved by the Ethics Committee of Huangshi Disease Prevention and Control Center.

The manifestations of pneumoconiosis on X-ray chest radiographs can be categorized into three stages. Stage I pneumoconiosis is characterized by small opacities with an overall profusion of category 1, distributed over at least 2 lung zones. Stage II pneumoconiosis is indicated by small opacities with an overall profusion of category 2, extending beyond 4 lung zones; or small opacities with an overall profusion of category 3, distributed across 4 lung zones. Stage III pneumoconiosis is defined by one of the following conditions: the presence of large opacities with a long axis of at least 20 mm and a short axis of at least 10 mm; small opacities with an overall profusion of category 3, extending beyond 4 lung zones with coalescence of opacities; or small opacities with an overall profusion of category 3, extending beyond 4 lung zones along with the presence of large opacities [16].

### Survival analyses

We expressed the categorical variables as frequencies and percentages (%). The chi-square test was used to compare the differences among categorical variable groups, while Analysis of Variance (ANOVA) was employed to assess differences among groups for continuous variables. Survival time was demarcated as the duration from the first diagnosis to either the date of death or the end of the study. Further scrutinizing survival across temporal cohorts within this study population, we employed a life table methodology. This analysis considered various age brackets at the time of initial diagnosis, with an initiation age of 25 and ≥ 70 years, segmented by five-year intervals.

For the detailed survival analysis, we applied the Kaplan-Meier method for the subgroup evaluations and Cox proportional hazards regression models to examine risk factors. The selection of variables for these models was grounded on findings from prior research, with a preliminary univariate Cox proportional hazards model screening where variables showing *P* < 0.2 were considered for further analysis. These selected variables were subsequently incorporated into a multivariate Cox proportional hazards model, focusing on pneumoconiosis patient survival time as the dependent variable. This was done with the aim of pinpointing significant risk factors influencing patient survival.

Model discrimination was assessed by utilizing receiver operating characteristic (ROC) curves, substantiated by evaluating the area under the curve (AUC), to reflect the distinct capabilities of the model based on subject work characteristics. Concurrently, calibration of the nomograms for the study cohort underwent graphical assessment through the deployment of calibration plots. The Brier score, serving as a precise metric for calibration curve performance, indicates higher model accuracy with decreasing values. A Brier score ranging from 0 to 0.1 denotes an excellent model, whereas scores from 0.1 to 0.25 are generally recognized as good. Comprehensive analyses were executed using R software version 4.3.1 (R Foundation for Statistical Computing, Vienna, Austria). The *P* < 0.05 was considered significant.

### Estimating DALYs

The DALY metric quantitatively measures disease burden by aggregating years of life lost (YLL) due to premature mortality and years lived with disability or disease (YLD), such that DALY = YLL + YLD. According to the method provided by the World Health Organization (WHO) [17], we chose not to discount YLLs, YLDs, or DALYs for time:

1 $${\rm{YL}}{{\rm{L}}_{{\rm{(c,s,a,t)}}}}{\rm{ = }}{{\rm{N}}_{{\rm{(c,s,a,t)}}}}{\rm{ \times }}{{\rm{L}}_{{\rm{(s,a)}}}}$$

where: N_(c, s,a, t)_ is the number of deaths due to the cause c for the given age (a) and sex (s) in year (t). L_(s, a)_ is a standard loss function specifying years of life lost for a death at age (a) for sex (s). The life expectancy at different age groups is based on the abridged life tables released by the Department of Economic and Social Affairs Population Division, with data sources as follows: https://population.un.org/wpp/Download/Standard/Mortality/.

2 $${\rm{YL}}{{\rm{D}}_{{\rm{(c,s,a,t)}}}}{\rm{ = }}{{\rm{I}}_{{\rm{(c,s,a,t)}}}}{\rm{ \times }}{{\rm{D}}_{{\rm{(c,s,a)}}}}{\rm{ \times }}{{\rm{L}}_{{\rm{(c,s,a,t)}}}}$$

where: I_(c, s,a, t)_ is the incidence (new cases) for a specific cause (c), sex (s), age group (a), and time period (t). D_(c, s, a)_ is the disability weight (DW) assigned to the condition for a specific cause (c), sex (s), and age group (a). The DW for pneumoconiosis is deemed equivalent to chronic obstructive pulmonary disease (COPD), with values of 0.019 for stage I, 0.225 for stage II, and 0.408 for stage III [11]. L_(c, s,a, t)_ is the average duration of the condition (in years) for a specific cause (c), sex (s), age group (a), and time period (t).

## Result

### Cohort assembly and profiling of study articipants

**Fig. 1:**
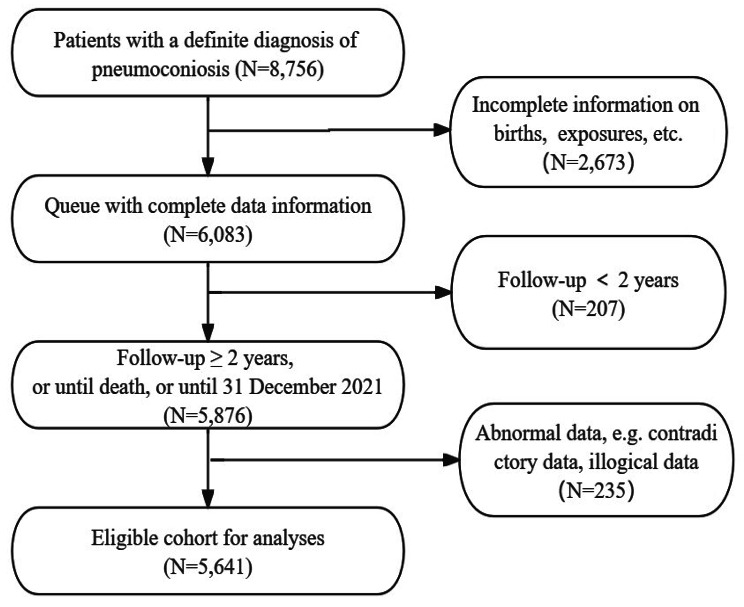
Flow diagram of the study population

We collected data on a cohort of 8,756 pneumoconiosis patients in Huangshi city, Hubei Province, from 1958 to 2021. In constructing the cohort, we first eliminated patients with incomplete data, especially those instances lacking critical birth details and exposure history (*N* = 2,673). Subsequently, we removed 207 cases that did not meet the minimum follow-up requirement of two years, as well as 235 patients who were excluded due to data inconsistencies, such as contradictory or illogical information. The remaining data of eligible patients were then incorporated into the definitive study cohort (*N* = 5,641) (Fig. 1).

### Demographic and clinical characteristics of pneumoconiosis patients

This study encompassed a cohort of 5,641 patients diagnosed with pneumoconiosis from Huangshi city, spanning from 1958 to 2021. Of these, a total of 2,388 fatalities were recorded. The period between 2000 and 2019 witnessed the greatest surge in both new cases and deaths, accounting for 3,807 and 2,317 respectively. Within the cohort, the prevalence of stage I pneumoconiosis notably high (92.34%), and the elderly population, aged 65 and above, represented 14.47% of this group. A temporal shift in the cohort’s entry point was observed to influence both the average duration of dust exposure and the age at initial diagnosis. The cohort members who joined between 2000 and 2019 presented the highest figures in these categories, averaging 23.53 years for dust exposure and 57.74 years for age at first diagnosis. (Table S1).

As delineated in Table 1, the patients within this pneumoconiosis study predominantly fell into stage I of the disease. Across the three delineated stages of pneumoconiosis, the frequency of male patients was substantially greater than that of females. When examining the types of pneumoconiosis among patients, coal workers’ pneumoconiosis was the most prevalent, documented in 4,027 cases (71.4%). This was followed by silicosis, recorded in 1,470 cases (26.1%). The age group from 45 to 59 years old represented the majority at first diagnosis, encompassing 51.9% of the patients. Notably, with 15–24 years of experience in dust exposure made up the largest portion with 2,667 or 47.3% of the total number of patients. All variables were statistically significant (*P* < 0.001) at all three stages except gender.

**Table 1:** Demographic and occupational characteristics

Characteristic	Stage I (*N* = 5,208)	Stage II (*N* = 356)	Stage III (*N* = 77)	*P*
Gender, n (%)				0.264 ^c^
Male	5,155 (98.98%)	355 (99.72%)	77 (100.00%)	
Female	53 (1.02%)	1 (0.28%)	0 (0.00%)	
Type, n (%)				0.007 ^c^
CWP ^a^	3,685 (70.76%)	285 (80.06%)	57 (74.03%)	
Silicosis	1,382 (26.54%)	69 (19.38%)	19 (24.68%)	
Cement pneumoconiosis	123 (2.36%)	1 (0.28%)	1 (1.30%)	
Others ^b^	18 (0.35%)	1 (0.28%)	0 (0.00%)	
Age at first diagnosis (years), n (%)	54.25 ± 10.60	51.96 ± 8.75	55.04 ± 7.96	< 0.001 ^d^
≤ 44	1,127 (21.64%)	80 (22.47%)	11 (14.29%)	< 0.001 ^c^
45–59	2,652 (50.92%)	230 (64.61%)	44 (57.14%)	
≥ 60	1,429 (27.44%)	46 (12.92%)	22 (28.57%)	
Dust exposure years (years), n (%)	22.49 ± 7.67	18.08 ± 8.20	16.18 ± 9.57	< 0.001 ^d^
≤ 14	884 (16.97%)	125 (35.11%)	39 (50.65%)	< 0.001 ^c^
15–24	2,481 (47.64%)	163 (45.79%)	23 (29.87%)	
≥ 25	1,843 (35.39%)	68 (19.10%)	15 (19.48%)	

^a^: Coal workers’ pneumoconiosis. ^b^: Other types of pneumoconiosis include: graphite pneumoconiosis, carbon black pneumoconiosis, asbestosis, talc pneumoconiosis, pottery worker’s pneumoconiosis, aluminosis, other pneumoconiosis, pneumoconiosis (unknown). ^c^: The *P*-value less than 0.05 in the chi-square test is indicative of a statistically significant difference. ^d^: The *P*-value less than 0.05 in the ANOVA is indicative of a statistically significant difference

### Survival analyses of pneumoconiosis patients

**Fig. 2:**
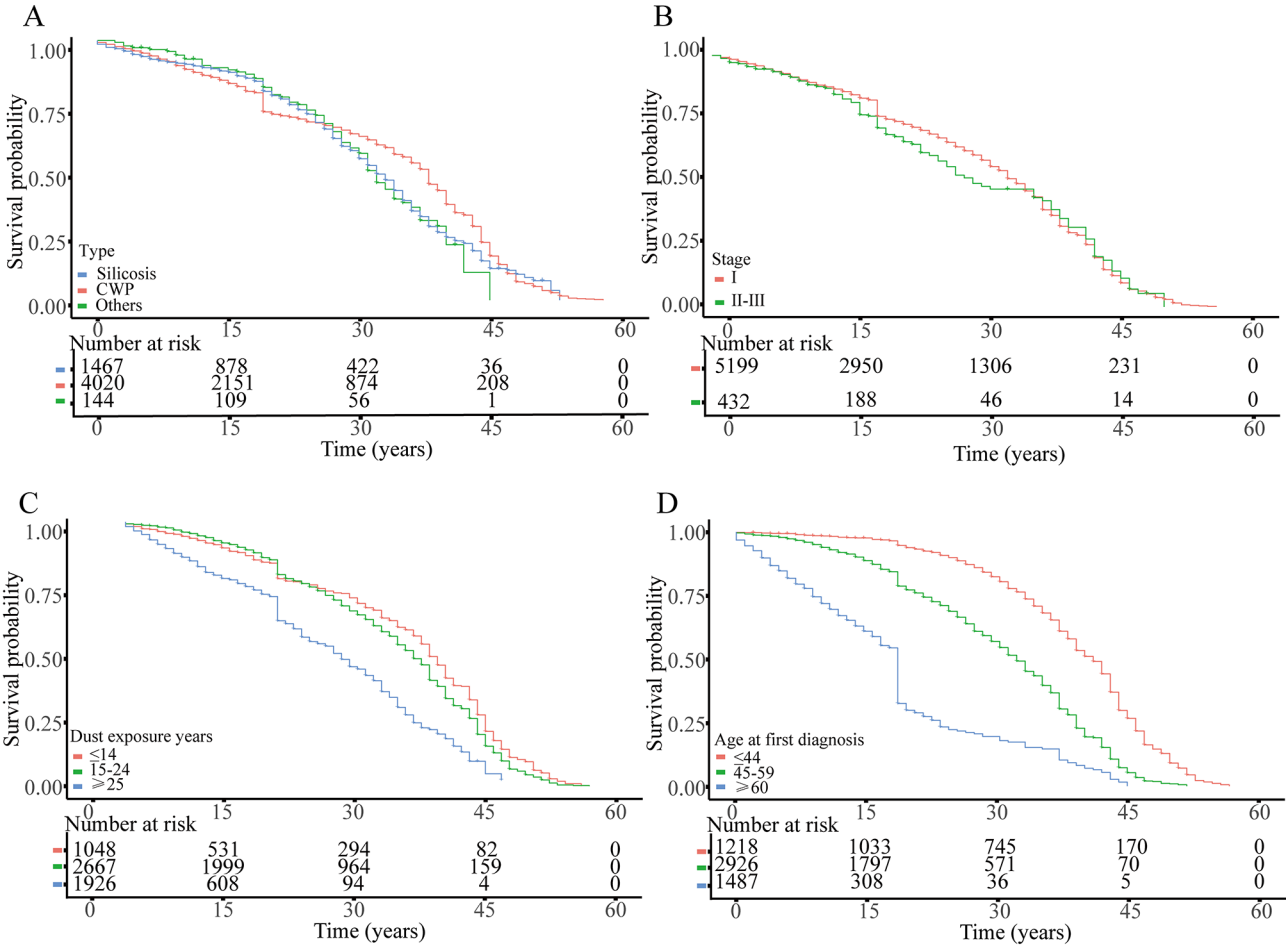
Survival curves of 5,641 pneumoconiosis patients across various cohorts in Huangshi city, Hubei Province. The cohorts were categorized based on the following criteria: (**A**) the type of pneumoconiosis; (**B**) the stage of pneumoconiosis; © the duration of dust exposure; (**D**) the age at initial diagnosis. CWP, coal workers’ pneumoconiosis

Table 2 presents the probability death and hazard ratios for pneumoconiosis across different age groups at first diagnosis, split into 5-year increments. An upward trend in mortality risk was noted (0.002–0.122), with hazard ratios also rising with age (0.001–0.28). Table S2 breaks down case-fatality rates and median survival by stage (I, II, III) and age subgroup, revealing shorter survival among older patients and median survival times being 18, 14, and 9 years across stages, respectively. Using the Kaplan-Meier method, estimated survival of the 5,641 pneumoconiosis patients in Huangshi city from 1958 to 2021. Survival differences across the three pneumoconiosis types were not significant (*P* = 0.121). Similarly, there was no notable survival difference when comparing stages I and II-III at first diagnosis (*P* = 0.283). However, patients with ≥ 25 years of dust exposure had significantly lower survival compared to others (*P* < 0.05). Furthermore, survival varied significantly with age at diagnosis: those ≤ 44 years had the best prognosis, while survival decreased for those 45–49 years, and further for those ≥ 60 years (*P* < 0.05) (Fig. 2).

**Table 2:** Cumulative survival rate and probability of death in 5,641 patients with pneumoconiosis: grouping by age of first diagnosis

Age Groups	Observed patients	Surviving patients	Died patients	Cumulatively observed patients	Corrected observed patients	Fatality rate (%)	Survival rate	cumulatively survival rate	Cumulatively fatality rate (%)	Death probability	Hazard ratio*
25 ~ 29	22	12	10	5,641	5,635.0	0.001	0.999	0.999	0.001	0.002	0.00
30 ~ 34	86	45	41	5,619	5,596.5	0.01	0.99	0.99	0.01	0.01	0.01
35 ~ 39	314	144	170	5,533	5,461.0	0.03	0.97	0.96	0.04	0.03	0.03
40 ~ 44	620	323	297	5,219	5,057.5	0.06	0.94	0.90	0.10	0.06	0.06
45 ~ 49	892	478	414	4,599	4,360.0	0.09	0.91	0.82	0.18	0.07	0.10
50 ~ 54	1,080	680	400	3,707	3,367.0	0.12	0.88	0.72	0.28	0.10	0.13
55 ~ 59	976	620	356	2,627	2,317.0	0.15	0.85	0.61	0.39	0.11	0.17
60 ~ 64	730	477	253	1,651	1,412.5	0.18	0.82	0.50	0.50	0.11	0.20
65 ~ 69	461	270	191	921	786.0	0.24	0.76	0.38	0.62	0.12	0.28
≥ 70	460	204	256	460	358.0	0.72	0.28	0.11	0.89	0.00	0.00

*: Hazard ratio(t) = f(t)/S(t)

The probability density function (PDF) is denoted by f(t) in survival analysis and it describes the probability per unit time of an event occurring at exactly time t

S(t) represents the survival function, which is the probability that the time to an event (such as death) is longer than t

### Cox proportional hazards analyses of pneumoconiosis patients

The model examined six factors for pneumoconiosis survival: dust exposure duration, age at first diagnosis, stage of disease, type of pneumoconiosis, medical insurance, and industry. Through the univariate cox model results, variables with *P* < 0.2 were included in the multivariate cox model, which included four variables: dust exposure years, age at first diagnosis, stage of disease, and type of pneumoconiosis. The multivariate cox model identified significant risk factors affecting survival time with statistical significance (*P* < 0.05): Dust exposure years (HR = 1.197, 95% CI: 1.104–1.298), age at first diagnosis (HR = 3.149, 95% CI: 2.961–3.349), silicosis (HR = 1.378, 95% CI: 1.254–1.515), other types of pneumoconiosis (1.456, 95% CI: 1.148–1.848) (Table 3).

**Table 3:** Cox regression analysis in 5641 patients with pneumoconiosis

Variables	Univariate Cox regression			Multivariate Cox regression		
	*β*	*P*	HR (95% CI) ^a^	*β*	*P*	HR (95% CI)
Dust exposure time (years)	0.541	< 0.001	1.717 (1.582, 1.864)	0.016	< 0.001	1.197 (1.104, 1.298)
Age at first diagnosis (years)	1.108	< 0.001	3.026 (2.851, 3.211)	0.082	< 0.001	3.149 (2.961, 3.349)
Types						
CWP ^a^	Ref					
Silicosis pneumoconiosis	0.142	0.004	1.152 (1.052, 1.263)	0.321	< 0.001	1.378 (1.254, 1.515)
Others	0.180	0.727	1.203 (0.943, 1.524)	0.379	0.002	1.456 (1.148, 1.848)
Stage						
I	Ref					
II-III	0.092	0.191	1.096 (0.938, 1.298)	0.249	0.004	1.282 (1.108, 1.517)
Medical insurance						
No	Ref					
Yes	-0.324	0.388	0.723 (0.501, 1.002)	-	-	-
Industries						
Mining	Ref					
Manufacturing	-0.196	0.307	0.822 (0.547, 1.236)	-	-	-
Others	0.219	0.377	1.245 (0.758, 2.044)	**-**	**-**	**-**

CWP: Coal workers’ pneumoconiosis; ^a^: 95% confidence interval

**Fig. 3:**
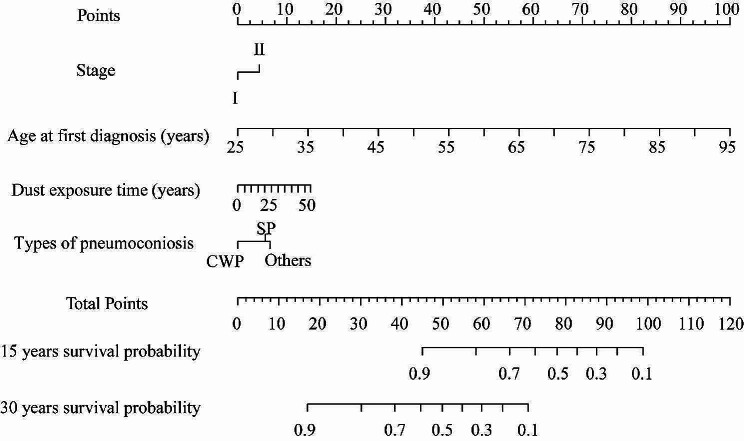
The nomogram of multivariate Cox proportional hazards model

**Fig. 4:**
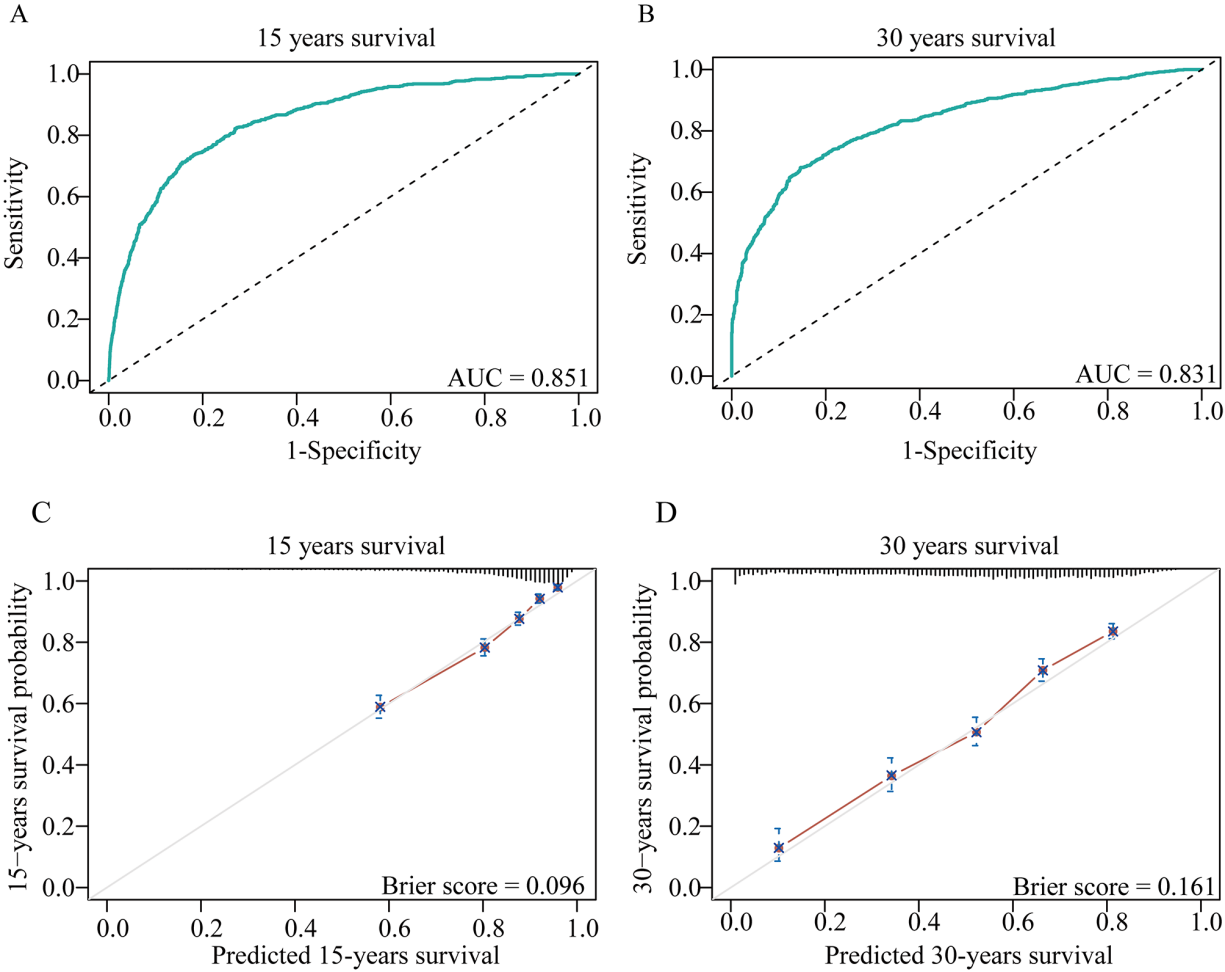
The ROC curve and calibration plot of the nomogram. (**A**) and (**B**) indicate the ROC curve and AUC of the nomogram in predicting 15- and 30-years survival. The calibration plot for predicting 15-years (**C**) and 30-years (**D**) survival. Actual rate of survival is shown on the y-axis, and the nomogram-predicted probability of survival is shown on the x-axis. ROC, receiver operating characteristic; AUC, area under the ROC curve

Utilizing the multivariate model, nomogram plots were created, and risk factor scores were aggregated to predict 15-years and 30-years survival probability; older age at diagnosis was especially predictive (Fig. 3). ROC and calibration curves for 15-years and 30-years survival showed good model discrimination with AUC (0.851 and 0.831). Calibration additionally showed a close concordance between predicted and observed survival, with Brier scores of 0.096 at 15 years and 0.161 at 30 years, confirming the model’s calibration (Fig. 4).

### Competing risks models of pneumoconiosis patients

The study employed the competing risks models to assess varying causes of death and conducted sensitivity analyses to confirm the reliability of the multivariate Cox model findings. The analysis indicated that factors such as age at first diagnosis, dust exposure time, silicosis, and stages II-III consistently emerged as potential risk factors in pneumoconiosis, evidencing the model’s stability (Table S3).

### Disease burden of pneumoconiosis

Between 1958 and 2021 in Huangshi city, the total DALYs were 7,974.35 person-years, with YLDs of 3,753.37 person-years and YLLs of 4,220.98 person-years. Table 4 details the disease burden across different pneumoconiosis features. In terms of total DALYs, the following were observed for each pneumoconiosis condition: silicosis accounted for 1,863.00 person-years, CWP for 5,894.22 person-years, cement pneumoconiosis for 192.51 person-years, and other types of pneumoconiosis for 24.62 person-years. CWP represented the highest burden in total DALYs. However, when assessing the average burden per individual case (average DALYs), cement pneumoconiosis was found to be higher than the other types.

**Table 4:** Disease burden of pneumoconiosis patients with different characteristics

Variables	Number	Death	DALY	YLD	YLL	Average DALY	Average YLD	Average YLL
Gander								
Male	5,587	2,360	7931.47	3,727.75	4,203.72	1.42	0.67	1.78
Female	54	28	42.88	25.61	17.26	8.89	8.54	0.62
Type								
Silicosis	1,471	656	1,863.00	875.05	987.95	1.27	0.59	1.51
CWP	4,026	1,660	5,894.22	2,721.45	3,172.77	1.46	0.68	1.91
Cement pneumoconiosis	125	59	192.51	146.19	46.32	1.54	1.17	0.79
Others ^a^	19	13	24.62	10.68	13.94	1.30	0.56	1.07
Age at first diagnosis (years)								
≤ 44	1,218	609	2,000.52	1,205.28	795.25	1.64	0.99	1.31
45–59	2,926	1,126	3,920.79	2,059.26	1,861.53	1.34	0.70	1.65
≥ 60	1,497	653	2,053.04	488.84	1,564.20	1.37	0.33	2.40
Stage								
I	5,209	2,242	5,732.19	2,040.49	3,691.70	1.10	0.39	1.65
II	356	116	1,694.78	1,351.80	342.98	4.76	3.80	2.96
III	76	30	547.39	361.08	186.31	7.20	4.75	6.21
Total	5,641	2,388	7,974.35	3,753.37	4,220.98	1.41	0.67	1.77

CWP: Coal workers’ pneumoconiosis; DALY: Disability-Adjusted Life Year; YLD: Years lived with disability; YLL: Years of life lost due to premature mortality; ^a^: Other types of pneumoconiosis include: graphite pneumoconiosis, carbon black pneumoconiosis, asbestosis, talc pneumoconiosis, pottery worker’s pneumoconiosis, aluminosis, other pneumoconiosis, pneumoconiosis (unknown)

Patients first diagnosed at age ≤ 44 years had the highest average DALYs (1.64 person-years), YLDs (0.99 person-years), and YLLs (1.31 person-years) among the age groups. While stage I posted the highest accumulated DALYs at 5,732.19 person-years, the average burden per case for stage III surpassed both stages I and II. Moreover, the elevated average YLLs for stage III, at 6.21 person-years, underscores the grave severity of pneumoconiosis in its advanced stages.

While comparing the disease burden over various epochs, it becomes apparent that the period from 2000 to 2019 witnessed the most substantial impact, characterized by the peak in both overall and average DALYs. This surge is attributed to the significant number of individuals diagnosed within this timeframe. Moreover, the decade spanning 1980 to 1999 saw a notably higher average in YLLs when contrasted with other periods (Table S4).

## Discussion

Pneumoconiosis poses a serious threat to the health of workers and places a significant burden on society, affecting millions of workers worldwide who engage in hazardous occupations [18]. It is one of the common occupational diseases in China. The primary findings of the retrospective cohort study on occupational pneumoconiosis conducted in Huangshi city, China from 1958 to 2021 reveal significant insights into the evolution of this occupational disease. The study indicates a distinct trend of decreasing survival rates among patients diagnosed with pneumoconiosis, reflecting prolonged exposure to hazardous materials without adequate protective measures. Additionally, the burden of disease, measured in terms of morbidity and mortality, has shown a substantial increase, highlighting the severe impact on workers’ health and the strain on healthcare systems. The data emphasize the critical need for improved occupational safety standards and health monitoring practices to prevent the onset and progression of pneumoconiosis among workers in industrial settings.

Previous studies indicated that the regional distribution of occupational pneumoconiosis is closely tied to the structure of industry [19]. The three most common types of pneumoconiosis included asbestosis, silicosis and CWP. Specifically, silicosis is more prevalent than asbestosis in developing countries, whereas in developed nations, the situation is reversed [20, 21]. According to China’s national occupational disease report covering the years 2015 to 2016, CWP and silicosis were listed as the top two reported new cases of pneumoconiosis [19]. Huangshi city has abundant coal resources, so most of the pneumoconiosis patients in this study were coal miners, followed by silicosis patients. This is different from the distribution of the main types of pneumoconiosis in Guangdong Province [22], Xinjiang Uygur Autonomous Region [23], and Tianjin [24]. For example, silicosis ranks first in Guangdong Province, followed by welders’ pneumoconiosis and other types of pneumoconiosis. Zhejiang Province mainly has non-ferrous and non-metallic mineral resources, so there are more cases of silicosis. Stage I (92.34%) still accounts for the majority of pneumoconiosis patients, followed by stage II and III, which is consistent with the characteristics of pneumoconiosis reported in Shanghai [5] and Tianjin [24].

Compared with stages I and II, the survival time of patients at stage III is much shorter, which is also consistent with previous studies [25–27]. This is because pneumoconiosis is a chronic disease with no effective cure, and its progression is accompanied by other complications and fibrosis of the lungs, leading to damage to lung function and affecting patient survival time [28]. It can be seen that prevention and control of disease progression is of great significance for improving the quality of life and survival time in patients with pneumoconiosis.

This study grouped pneumoconiosis patients with different characteristics and constructed their survival curves. In terms of different types of pneumoconiosis, this study found no significant differences in the survival curves among the three categories, which is consistent with a study conducted in Jiangsu Province [12]. However, we did not observe statistically significant differences in survival among different stages of pneumoconiosis in this study, which is inconsistent with a previous study conducted in Zhejiang Province [18]. This discrepancy in findings may be attributable to the smaller number of stage III patients in our study and the relatively similar survival status between stage II and stage I patients. Both the grouping of dust exposure years and the grouping of age at first diagnosis showed that the survival curves of the high-exposure group were lower than those of the low-exposure group, and the survival curves of the elderly group were lower than those of the young group. These results are consistent with previous studies, suggesting that prolonged exposure to dust and delayed diagnosis may both negatively impact the survival time of pneumoconiosis patients.

The Cox proportional hazards model analysis results of this study show that dust exposure time, first diagnosis age, silicosis, other types of pneumoconiosis, and stage II-III pneumoconiosis are important risk factors that affect patient survival time, which is similar to the results of other studies [29]. Dust exposure can influence patient mortality. This may be due to the cumulative effects of dust exposure from extended periods of working in industries with high dust levels. Relevant studies have also shown that even dust with relatively small damage to the human body can cause significant damage to patients’ health when the cumulative dust exposure is large [30]. The age of first diagnosis also affects pneumoconiosis patients’ survival to a certain extent. Patients diagnosed with pneumoconiosis at a younger age have better immunity, which is more conducive to preventing disease progression [31]. Patients with silicosis had a higher hazard ratio than those with CWP, which may be due to the higher content of free silica, which is more harmful to health. Relevant studies also show that patients with silicosis have a lower survival rate than other types of pneumoconiosis patients [32]. In addition, a diagnosis of stage II-III significantly impacts survival time compared to patients in stage I of the disease. Stage II-III pneumoconiosis represents a more advanced stage of disease progression, characterized by further pathological changes in the lungs as the stage of pneumoconiosis increases. The exacerbation of pulmonary fibrosis and more severe impairment of lung function have a direct impact on patients’ quality of life and overall survival [12]. Therefore, it is imperative to conduct regular health check-ups for the early detection of pneumoconiosis and to facilitate timely interventions. Doing so can prevent disease progression and enhance the treatment outcomes and prognosis for patients.

The cumulative DALYs of Huangshi city’s pneumoconiosis patients included in this study from 1958 to 2021 were 7,974.35 person-years, of which the cumulative YLDs were 3,753.37 person-years, and the cumulative YLLs were 4,220.98 person-years. The main contributor to the burden of pneumoconiosis in Huangshi city is the disability resulting from premature death, which contradicts the findings of previous studies [33]. This discrepancy can be attributed to the different weighting of pneumoconiosis disability in this study, considering various stages, as opposed to previous studies that used a fixed value, resulting in lower YLDs estimates. The mean YLLs in this study were 1.77 person-years, which was lower than the previous mean YLLs of 6.35 person-years in the Chinese pneumoconiosis population from 1990 to 2017. The mean YLDs were 0.67 person-years, lower than the 6.22 person-years in the previous study [34]. The average DALYs were higher in cement pneumoconiosis than in other types of pneumoconiosis, but the mean YLLs were higher in silicosis and coal miners’ pneumoconiosis than in cement pneumoconiosis. This may be due to the fact that silicosis and coal miners’ pneumoconiosis cause more severe damage to lung function, leading to premature death. In contrast, patients with cement pneumoconiosis may have slower disease progression, resulting in a higher YLDs. As the age of diagnosis increases, the average DALYs and average YLDs decrease. Early diagnosis of pneumoconiosis correlates with greater loss of healthy life years, as indicated by higher YLDs at younger ages. Conversely, older patients often exhibit weaker immune responses and overall health, heightening mortality risks. Furthermore, a diagnosis of pneumoconiosis at a later stage was found to be associated with increased average YLLs, which supports the COX proportional hazards model indicating that a more advanced initial stage reduces survival time in pneumoconiosis patients. Late diagnosis leads to higher mean YLLs due to disease progression and greater lung damage, resulting in a decreased life expectancy.

This study found that the highest disease burden, as measured by cumulative DALYs, occurred between 2000 and 2019, likely due to enhanced pneumoconiosis screening efforts, resulting in increased diagnosis of new cases. Advances in medical technology extended patient survival and decreased mortality, explaining the lower average YLLs in the 2000–2019 period compared to 1980–1999.

The study faces several limitations. Its retrospective nature introduces potential bias, and the absence of data on disease stage, patients’ economic status, and treatment costs impedes a full economic burden assessment. Besides, the lengthy time frame of the data and shifting testing criteria over different periods may impact the study’s findings.

## Conclusion

The historical retrospective cohort study conducted in Huangshi city, China, covering the years 1958–2021, has provided critical insights into the survival trends and disease burden of occupational pneumoconiosis. The findings reveal a significant impact on the affected populations, underscoring the urgency for enhanced preventive measures, early detection, and effective management strategies. This study not only highlights the persistent challenge of pneumoconiosis in industrial settings but also serves as a crucial call to action for policymakers, healthcare providers, and stakeholders to mitigate occupational health risks and improve the quality of life for individuals suffering from this debilitating condition. Furthermore, it underscores the importance of ongoing research and tailored interventions to address the evolving landscape of occupational health hazards.

### Electronic supplementary material

Below is the link to the electronic supplementary material.

Supplementary Material 1

## Data Availability

The datasets used during the current study available from the corresponding author on reasonable request (tonygqin@ntu.edu.cn).
