# Supplementary material for: Survival and disease burden analyses of occupational pneumoconiosis during 1958–2021 in Huangshi city, China: a retrospective cohort study
Source: BMC Public Health. 2024 May 29;24:1437. doi: 10.1186/s12889-024-18847-6 (PMC11137949; doi:10.1186/s12889-024-18847-6)
Supplement: Supplementary file 1 — Supplementary Material 1 [file 12889_2024_18847_MOESM1_ESM.docx]

**Supplementary Information**

**Survival and disease burden analyses of occupational pneumoconiosis during 1958-2021 in Huangshi city, China: A retrospective cohort study**

Hai-Lian Chen^1,2☯^, Chun-Hu Li^1☯^, Pei-Yao Zhai^3☯^, Xun Zhuang^4^, Yu-Long Lian^4^, Xue Qiao^2^, Jian Feng^5*^, Zu-Shu Qian^2*^, Gang Qin^1,5*^

^1^Joint Division of Clinical Epidemiology, Affiliated Hospital of Nantong University, School of Public Health of Nantong University, Nantong, Jiangsu, China

^2^Huangshi Center for Disease Control and Prevention, Huangshi, Hubei, China

^3^Department of Infectious Diseases, Affiliated Hospital of Nantong University, Medical School of Nantong University, Nantong, JS, China

^4^Department of Epidemiology and Biostatistics, School of Public Health of Nantong University, Nantong, Jiangsu, China

^5^National Key Clinical Construction Specialty-Department of Respiratory and Critical Care Medicine, Affiliated Hospital of Nantong University, Nantong, Jiangsu, China;

* tonygqin@ntu.edu.cn (GQ); 363530285@qq.com (ZSQ); Jfeng68@126.com (JF)

**Figure S1**


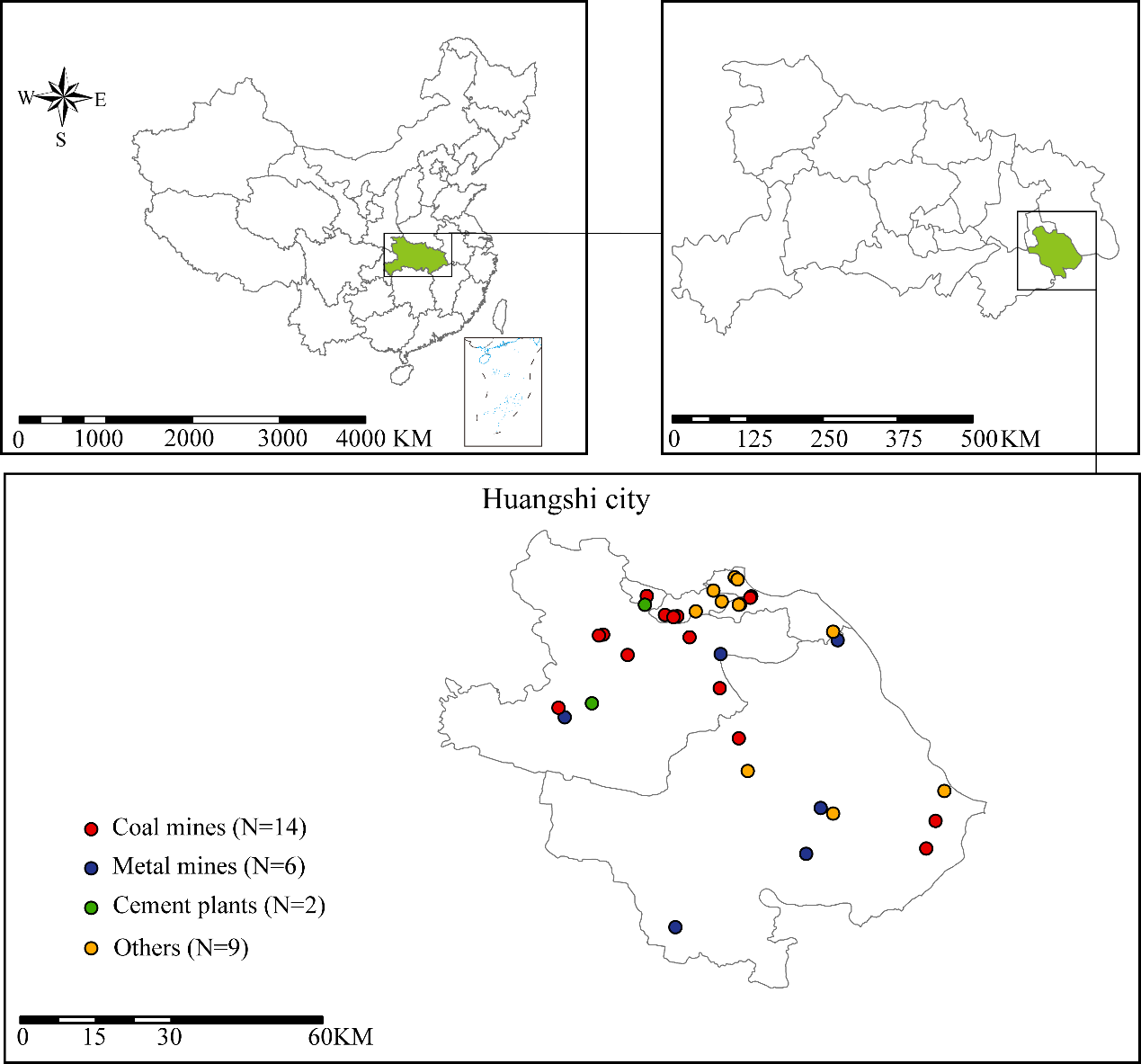


**Figure S1**. Huangshi city and its location in Hubei Province.

**Table S1**. Baseline data of the cohort of pneumoconiosis patients in Huangshi City, Hubei Province, 1958-2021.

| Year ^a^ | New patients | All patients | Death | Proportion stage Ⅰ (%) | Average Dust exposure years | Average Age at first diagnosis | Proportion of elderly patients (%) ^a^ |
| --- | --- | --- | --- | --- | --- | --- | --- |
| 1958-1970 | 769 | 769 | 0 | 94.80 | 18.48 | 43.65 | 0.39 |
| 1980-1990 | 1065 | 1834 | 71 | 96.71 | 19.72 | 48.72 | 2.82 |
| 2000-2019 | 3807 | 5570 | 2317 | 90.60 | 23.53 | 57.74 | 20.57 |
| Total | 5641 |  | 2388 | 92.34 | 22.12 | 54.12 | 14.47 |

^a^: This time is when the patient first entered the cohort, not the end of follow-up. ^b^: We defined patients over 65 years of age as elderly patients.

**Table S2.** Survival time of patients with pneumoconiosis in Huangshi City, Hubei Province, by age groups, 1958-2021

|  | Stage Ⅰ | | | | Stage Ⅱ | | | | Stage Ⅲ | | | |
| --- | --- | --- | --- | --- | --- | --- | --- | --- | --- | --- | --- | --- |
| Age Groups | Observed patients | Died patients | Fatality rate (%) | Median survival time(years) | Observed patients | Died patients | Fatality rate (%) | Median survival time(years) | Observed patients | Died patients | Fatality rate (%) | Median survival time(years) |
| 25~29 | 20 | 8 | 40.00 | 52 | 2 | 2 | 100.00 | 32 | 0 | 0 | 0.00 | - |
| 30~34 | 80 | 41 | 51.25 | 49 | 6 | 0 | 0.00 | - | 0 | 0 | 0.00 | - |
| 35~39 | 291 | 159 | 54.64 | 43 | 22 | 10 | 45.45 | 45 | 1 | 1 | 100.00 | 50 |
| 40~44 | 574 | 279 | 48.61 | 41 | 38 | 15 | 39.47 | 44 | 8 | 3 | 37.50 | 21 |
| 45~49 | 828 | 394 | 47.58 | 37 | 55 | 16 | 29.09 | 27 | 9 | 4 | 44.44 | 14 |
| 50~54 | 962 | 367 | 38.15 | 34 | 99 | 27 | 27.27 | 30 | 19 | 7 | 36.84 | 23 |
| 55~59 | 882 | 326 | 36.96 | 28 | 77 | 24 | 31.17 | 44 | 17 | 6 | 35.29 | 18 |
| 60~64 | 680 | 237 | 34.85 | 21 | 34 | 10 | 29.41 | 25 | 16 | 6 | 37.50 | 10 |
| 65~69 | 441 | 180 | 40.82 | 20 | 15 | 9 | 60.00 | 13 | 5 | 2 | 40.00 | 20 |
| ≥70 | 450 | 127 | 28.22 | 15 | 8 | 3 | 37.50 | 24 | 2 | 2 | 100.00 | 14 |
| Total | 5208 | 2241 | 43.03 | 18 | 356 | 116 | 32.58 | 14 | 77 | 31 | 40.26 | 9 |

**Table S3**. Competing risk model analysis in 5641 patients with pneumoconiosis.

| Variables | ALL ^a^  HR  (95%CI) | Lung cancer ^d^  HR  (95%CI) | Tuberculosis  HR  (95%CI) | Respiratory infections  HR  (95%CI) |
| --- | --- | --- | --- | --- |
| Dust exposure time | 1.197  (1.104, 1.298) | 1.005  (1.001，1.013) | 1.012  (1.003, 1.020) | 1.003  (0.995, 1.012) |
| First diagnosis of age | 3.149  (2.961, 3.349) | 1.073  (1.067, 1.078) | 1.078  (1.072, 1.084) | 1.059  (1.054, 1.065) |
| Type |  |  |  |  |
| CWP ^b^ |  | Ref | |  |
| Silicosis | 1.378  (1.254, 1.515) | 1.294  (1.175, 1.425) | 1.362  (1.242, 1.494) | 1.057  (0.948, 1.180) |
| Others ^c^ | 1.456  (1.148, 1.848) | 1.090  (0.801, 1.485) | 1.475  (1.218, 1.787) | 1.242  (0.993, 1.553) |
| Stage |  |  |  |  |
| Ⅰ |  | Ref | |  |
| Ⅱ-Ⅲ | 1.282  (1.108, 1.517) | 1.173  (0.962, 1.432) | 1.288  (1.066, 1.555) | 1.296  (1.091, 1.539) |

^a^ is to include outcomes other than pneumoconiosis, including lung cancer, tuberculosis, respiratory infections, cardio-cerebrovascular diseases, as competing risks. ^b^: Coal workers' pneumoconiosis. ^c^: Other types of pneumoconiosis include: graphite pneumoconiosis, carbon black pneumoconiosis, asbestosis, talc pneumoconiosis, pottery worker’s pneumoconiosis, aluminosis, other pneumoconiosis, pneumoconiosis (unknown). d Lung cancer outcome enters the model as a risk competing event.

**Table S4**. Disease burden caused by pneumoconiosis in different years in Huangshi city, Hubei Province.

| Year | Number | Death | DALY | YLD | YLL | Average DALY | Average YLD | Average YLL |
| --- | --- | --- | --- | --- | --- | --- | --- | --- |
| 1958-1970 | 769 | 0 | 105.32 | 105.32 | 0.00 | 0.14 | 0.14 | 0.00 |
| 1980-1999 | 1834 | 71 | 1229.79 | 798.70 | 431.09 | 0.67 | 0.44 | 6.16 |
| 2000-2019 | 5570 | 2317 | 6639.24 | 2849.35 | 3789.89 | 1.19 | 0.51 | 1.64 |

^a^: This time is when the patient first entered the cohort, not the end of follow-up.
